# Volition-adaptive control for gait training using wearable exoskeleton: preliminary tests with incomplete spinal cord injury individuals

**DOI:** 10.1186/s12984-017-0345-8

**Published:** 2018-01-03

**Authors:** Vijaykumar Rajasekaran, Eduardo López-Larraz, Fernando Trincado-Alonso, Joan Aranda, Luis Montesano, Antonio J. del-Ama, Jose L. Pons

**Affiliations:** 1 School of Computer Science, University of Birmingham, Birmingham, UK; 2 Institute of Medical Psychology and Behavioral Neurobiology, University of Tübingen, Tübingen, Germany; 3 Biomechanics and Technical Aids unit, National Hospital for Spinal cord injury, Toledo, Spain; 4 Departamento de Automatic e Control, Universitat Politécnica de Catalunya, Barcelona-Tech, Barcelona, Spain; 5 Departamento de Informatica e Ingenería de Sistemas and, Instituto de Investigacion en Ingeneria de Aragon (I3A), University of Zaragoza, Zaragoza, Spain; 6 Neural Rehabilitation group, Spanish National Research Council (CSIC), Cajal Institute, Madrid, Spain

**Keywords:** Adaptive control, Brain-machine interface, Exoskeleton, Finite-state machine, Gait training, Incomplete SCI, Volitional control

## Abstract

**Background:**

Gait training for individuals with neurological disorders is challenging in providing the suitable assistance and more adaptive behaviour towards user needs. The user specific adaptation can be defined based on the user interaction with the orthosis and by monitoring the user intentions. In this paper, an adaptive control model, commanded by the user intention, is evaluated using a lower limb exoskeleton with incomplete spinal cord injury individuals (SCI).

**Methods:**

A user intention based adaptive control model has been developed and evaluated with 4 incomplete SCI individuals across 3 sessions of training per individual. The adaptive control model modifies the joint impedance properties of the exoskeleton as a function of the human-orthosis interaction torques and the joint trajectory evolution along the gait sequence, in real time. The volitional input of the user is identified by monitoring the neural signals, pertaining to the user’s motor activity. These volitional inputs are used as a trigger to initiate the gait movement, allowing the user to control the initialization of the exoskeleton movement, independently. A Finite-state machine based control model is used in this set-up which helps in combining the volitional orders with the gait adaptation.

**Results:**

The exoskeleton demonstrated an adaptive assistance depending on the patients’ performance without guiding them to follow an imposed trajectory. The exoskeleton initiated the trajectory based on the user intention command received from the brain machine interface, demonstrating it as a reliable trigger. The exoskeleton maintained the equilibrium by providing suitable assistance throughout the experiments. A progressive change in the maximum flexion of the knee joint was observed at the end of each session which shows improvement in the patient performance. Results of the adaptive impedance were evaluated by comparing with the application of a constant impedance value. Participants reported that the movement of the exoskeleton was flexible and the walking patterns were similar to their own distinct patterns.

**Conclusions:**

This study demonstrates that user specific adaptive control can be applied on a wearable robot based on the human-orthosis interaction torques and modifying the joints’ impedance properties. The patients perceived no external or impulsive force and felt comfortable with the assistance provided by the exoskeleton. The main goal of such a user dependent control is to assist the patients’ needs and adapt to their characteristics, thus maximizing their engagement in the therapy and avoiding slacking. In addition, the initiation directly controlled by the brain allows synchronizing the user’s intention with the afferent stimulus provided by the movement of the exoskeleton, which maximizes the potentiality of the system in neuro-rehabilitative therapies.

## Background

Neurological disorder affects the individuals in performing their activities for daily living and to constantly rely on external support. Specifically gait deficit is one of the challenges faced by the individuals affected by any neurological disorder such as Spinal cord injury (SCI) or stroke. Gait training for patients with neurological disorders is of great interest for the researchers involved in developing such assistive technologies. However, the nature and level of neurological disorder challenges the development of a universally viable assistance [1, 2]. Hence, a user specific assistance model is needed to provide the suitable gait compensation and to ensure equilibrium. An efficient control strategy must consider the physical interaction of the user and signal-level feedbacks during the practical use [3, 4]. The effectiveness of the control model can also be enhanced by combining multiple therapeutic modalities to promote an engaging and challenging training, following with more naturalistic movements [5]. Several treadmill-based training robots such as Lokomat [6], LOPES [7–9], have proven to be efficient in providing the necessary gait assistance to the users, especially for paraplegic patients. The availability of an external body support system handles the complications in maintaining the equilibrium [8, 10–12], whilst lack of active in-therapy involvement of the user which frequently leads to slacking in patients [13]. Hence, these devices are beneficial for individuals with complete Spinal cord injury (SCI) or acute Stroke, possessing less muscular strength to perform a movement [11]. In wearable robots, gait assistance is challenging in determining the level of assistance for ensuring dynamic stability, considering the ground reaction forces acting on them BLEEX [14], XPED2 [15], Ekso (earlier eLegs) [16], Rex (Rex Bionics) and Re-Walk [17]. These exoskeletons have proven to be capable in providing assistance on a passive range of motion and using complex systems [4].

Assistance in robotic rehabilitation can be achieved using an effective control strategy [18, 19] such as impedance or adaptive control, which acts based on the subjects’ performance. Such control strategies operate under the principle of assistance-as-needed, in which assistive forces increase as the participant deviates from the desired trajectory [20]. The deviation of the user can also be used as an input to generate a trigger to initiate the movement or the assistance in accordance to the users’ performance [21]. For gait, a personalized assistance based on the user’s intentions and movements is needed to dynamically adapt to the users’ needs. A predefined trajectory pattern based control, without other inputs, imposes a complete assistance which might induce slacking and harm the patient [18]. Thus, it is necessary to measure the human-orthosis interaction torques, to evaluate the user performance and status, in order to design a hybrid combination of force-position control. A similar method by means of the application of torque based on the user interaction was evaluated using a wearable device [22–24], where the knee joint torque is applied by estimating the required joints’ muscle actuations. Another method of assistance by just applying torques in the joints has been demonstrated to provide assistive behaviour for stroke individuals, with the trajectories being enforced by the orthosis, Vanderbilt [25–27] and H2 [28]. A similar study, enforcing the symmetry based gait compensation in stroke individuals has been performed using HAL, based on the movement of the unaffected limb. Such symmetry based adaptation considers assistance for the swing phase on the basis of the data compensated by the user [29, 30]. In case of incomplete SCI individuals, this type of assistance cannot be viable, considering that the major concern is in maintaining balance [31].

The combination of muscle synergies and neural signals for evaluating the volitional commands of the patient has gained more importance in the recent years, as a top-down approach in rehabilitation [32]. The detection of the best instant for gait initiation and termination was performed to develop a volitional control based robotic rehabilitation [22, 33]. This type of volitional input motivate the user to initiate the therapy and subsequently improves the ability of intention [34, 35]. User intention based rehabilitation can be implemented by different approaches such as using an external input order (joystick) [36], muscle activity [37], joint activity [38] or interaction/contact forces [39]. One of the widely used approaches for monitoring the human intention relies on the use of brain machine interfaces(BMI) such as in Lokomat [40, 41] XoR [42], Rex [43] and MINDWALKER [44]. These systems are efficient in monitoring the user intentions mainly, because a real displacement of the joint position is not needed always to initiate the gait.

This work presents an assistive control strategy for individuals with incomplete SCI, using a wearable robot, to perform user-dependent gait initiation and assistance, in real time. The electroencephalographic (EEG) signals are used to monitor the motor intention brain activity of the patients at the beginning of every gait cycle, in order to know if the patient is ready to start. This BMI-triggered gait initiation approach ensures user involvement in the therapy and their motivation in performing the task. Further, a Finite-state machine (FSM) based control model is used in this set-up which helps in combining these volitional orders with the gait adaptation. For gait assistance, the control model employs a variable impedance based approach, without neither treadmill training nor an external body weight support. The absence of weight compensation carries with it the challenging task of maintaining the equilibrium in presence of ground reaction forces. The adaptive control strategy employs joint interaction torques for evaluating and reconstructing the exoskeleton’s joint performance to provide sufficient assistance to the user. The goal of this work is to validate the proposed BMI-triggered adaptive control model for providing the necessary assistive behaviour to incomplete SCI individuals. The adaptive approach also enhances the patients’ gait performance by considering their interaction with the exoskeleton. This real time adaptation, based on the hybrid combination of joint position and interaction torques, ensures synchronization among the joint trajectories to maintain dynamic stability.

## Volitional-adaptive control

**Fig. 1:**
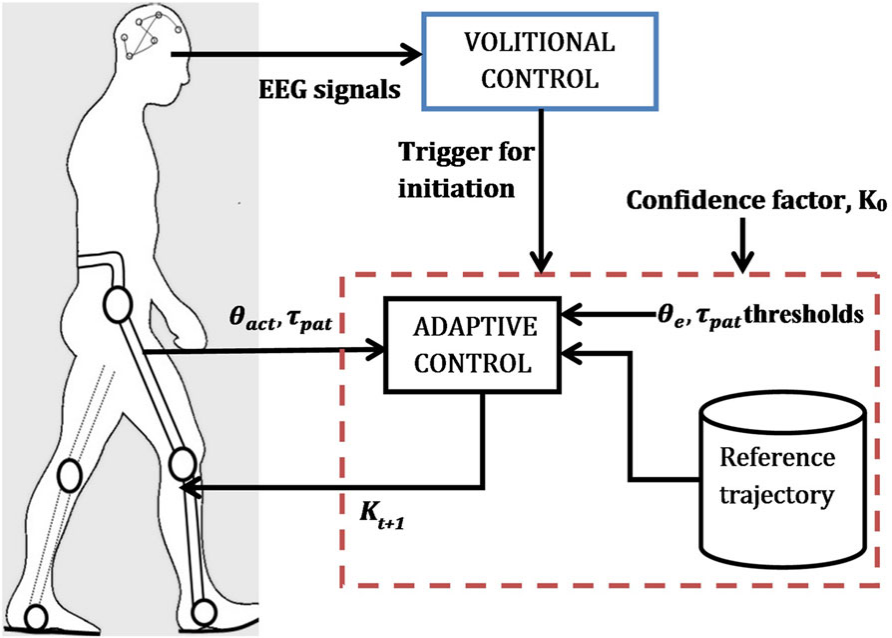
Schematic representation of the volition based adaptive control with BMI system and exoskeleton. BMI system triggers the adaptive control model which initiates the exoskeleton movement

A user specific control model can be realized by identifying the two major actions involved: intentions and movements of the user. User intentions or volitional orders can be recognized either by following the user movement in close contact (mechanical interaction) or by detecting the motor-related brain activity of the user. In this work, as shown in Fig. 1, the volitional control model involves only monitoring the neural signals using BMI, with the aim to determine the gait initiation. The adaptive control model involves monitoring the joint angles and human-orthosis interaction torques, from the exoskeleton and calibrating the input stiffness order to each joint, based on the user interaction. This volition based control exhibits an adaptive assistance to different pathologies and morphologies, which involves providing joint level assistance and ensuring dynamic stability.

The dynamic analysis of such a human centered adaptive control model must consider the human-orthosis interaction, in such a way that each joint actuator works in collaboration with the patient. The applied actuator torque can be modified by varying the joint stiffness parameter, which invariably modifies the corresponding joint trajectory and the force compensation (Fig. 1). This stiffness variation alters the actuator torque, which subsequently determines the degree of control transferred from the orthosis to the human or vice versa. Such an impedance control scheme has been widely used for its compliant behaviour, which results in an adaptive walking pattern and a more natural interaction between patient and orthosis [31, 45–48]. Thus, the impedance control can be determined by the following equation,

1 $$ F=M\ddot{\theta}+C\dot{\theta}+K\left(\theta_{ref}-\theta_{act}\right)  $$

where, *θ*_*ref*_ and *θ*_*act*_ are the reference and actual joint positions respectively, K is the stiffness parameter of the joint and F represents the applied force to the joint. M represents the mass, C is the damping constant and a and v represent the acceleration and velocity of the robot respectively. Since the terms M and C are uncontrollable and independent of the user performance, varying the stiffness parameter K is effectual for modifying the assistance or resistance to be provided. Hence the force equation, influenced by the stiffness parameter and position error, is represented as

2 $$ F=K\left(\theta_{ref}-\theta_{act}\right)  $$

The value of the stiffness (K) is determined dynamically for every joint, based on the performance of the user and the level of assistance to be exerted by the orthosis. It can be expressed as:

3 $$ K_{t+1}=K_{t} \pm \Delta K  $$

4 $$ \Delta K=\left|{\frac{\left(\theta_{ref}-\theta_{act}\right)}{s*max\left(\tau_{pat}\right)}}\right|  $$

where, max *τ*_*pat*_ is the vector of maximum values of human-orthosis interaction torques, registered during the initialization stage. s is a confidence factor (0.1 - 1) used to determine the applied stiffness at time t+1 and indeed, it is a vector of different confidence factor values specific to each joint. The confidence factor helps the therapist to individually assign the assistance level depending on the user’s disability. The term *ΔK* is a vector of stiffness variation to be applied to each joint, an incrementing or decrementing factor, obtained by comparing the interaction torques and position error, with respect to the predefined thresholds.

**Fig. 2:**
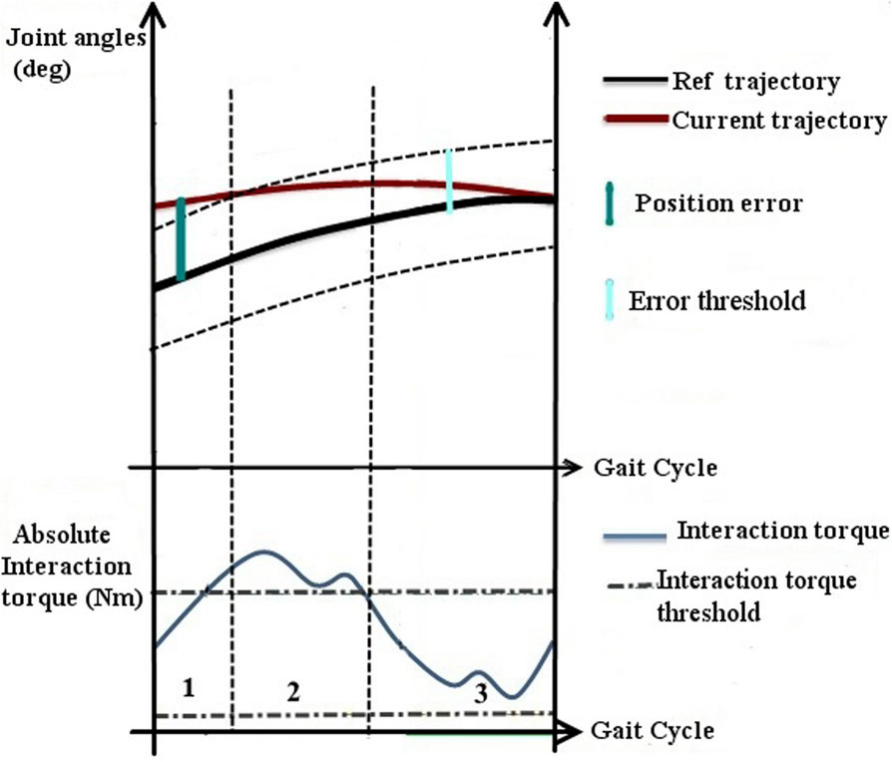
Relation between the stiffness variation and the input parameters: interaction torques and joint angles

Both, the confidence factor *s* and the initial stiffness *K*_*t*_ are variable parameters which shall be defined by the therapist in function of the capabilities of the patient. This confidence factor can be assigned with respect to the progress of the user, such as to modify the level of assistance provided. A low confidence factor (0.1) means that the user must be completely assisted while a higher confidence factor (0.9) indicates that the subject is capable of walking without or with little assistance.

Similarly, a variation of the *K* value results in a change in the force acting at the joint level which is perceived as assistance or resistance by the patient. Thus, the K value is incremented or decremented as a function of the evolution of the patient. The variation of the stiffness is categorized as three cases, as shown in Fig. 2;

1. When position error is over the threshold, then K must be incremented.
2. If the position error is less than the error threshold, but the interaction torque is over the corresponding threshold, then K must be maintained.
3. If position error is below the error threshold and the absolute interaction torque is low, then K must be decreased.

This adaptation can be employed in general, even in the presence of external perturbations. A detailed description of this stiffness adaptation model is available in [38, 49].

## Methods

The proposed volitional-adaptive gait assistance is based on monitoring the patients’ motor activity and their interaction with the orthosis. Hence, the experimentation section of this study involves two major systems: BMI and Exoskeleton. A protocol has been defined to perform the trials with incomplete SCI individuals. The performance of the adaptive gait assistance is demonstrated by comparing its results against those obtained by applying a constant impedance value. The metrics for evaluation relies only on the data provided by the exoskeleton, without the need of any external sensors.

### Participants

The presented study is performed with 4 incomplete SCI individuals with different morphological parameters, which helped in observing the diversified behaviour of the strategy. The demographic details about the participants are mentioned in Table 1. These incomplete SCI patients were selected at the Hospital Nacional de Parapléjicos, in Toledo (Spain), with gait prognosis and at the early stages of walking rehabilitation. Other requirements for the patients were to maintain balance between parallel bars; and to keep sufficient upper-limb strength to manage a walker or crutches, and to transfer from the wheelchair to a chair. All the subjects were informed about the study and a written consent was obtained before the session. The experimental study has been carried out after the formal approval of the local ethical committee of the hospital, Hospital Nacional de Parapléjicos-Toledo, Spain (C.E.I.C - 31/02/2014).

**Table 1:** Demographic details and ASIA impairment scale of the participants

	P01	P02	P03	P04
Age	30	24	49	21
Gender	M	M	F	M
Height (m)	1.85	1.92	1.6	1.8
Weight (kg)	90	57	76	57
Level of Lesion	L1	L1	T12	T11
Left leg Muscle test	8	13	6	9
(max 25)				
Right leg Muscle test	12	8	11	19
(max 25)				
ASIA ^*a*^	C	C	C	C
Time after injury	12	24	11	5
(months)				
Etiology	Trauma	Trauma	Trauma	Trauma

^*a*^ASIA-American Spinal Injury Association’s impairment assessment scale

### Protocol

The clinical protocol was defined based on the abilities of the patient and their conditions. The subjects performed an initial walking trial with a fixed stiffness and manual triggers as BMI inputs, to analyse the necessary walking assistance and to ensure that the patients get accustomed to the orthosis. An optimized gait pattern, obtained from the average of five healthy subjects, was used as a reference in the fixed stiffness mode. In the following sessions the patients were assisted based on the adaptive stiffness variation with respect to their interaction. In this case, the exoskeleton adapts to the patients’ pattern and varies the stiffness based on the interaction torques, while following the predefined trajectory. Hence, the walking experiment was performed in 3 sessions, performed in 3 consecutive days: Fixed stiffness (first session) and variable stiffness (next 2 sessions).

Each session lasted for a time span of 60-90 min, which includes the time for wearing the exoskeleton and the BMI calibration. Since the patients were not able to maintain their equilibrium for a long period of time, the joints of the exoskeleton were maintained rigid (90*Nm*/*deg* stiffness) during the BMI calibration. The fixed stiffness values were chosen heuristically based on the studies performed with healthy subjects. Based on these preliminary studies conducted with healthy subjects [38, 49], the initial stiffness and confidence factor for all the SCI patients and joints, for the adaptive stiffness study, were assumed to be 80Nm/deg and 0.6 respectively. The confidence factor and the stiffness values were also maintained the same for all the patients, considering their similar impairment level. We impose equal initial values, for all the joints and all the patients, to evaluate the adaptability of the algorithm independent of patients’ specific health condition. The number of gait cycles per session has been defined based on a probabilistic study by comparing the fixed and variable stiffness conditions. The sample size returned a power of 0.9852, with *z-test* probability method, for a minimum of 10-15 gait cycles. Hence, each session comprised of a maximum of 20 gait cycles, taking into account the physical level of the participants involved. Since some of the users were not comfortable with walkers and crutches, parallel bars were used as external support for this experimentation. A detailed study about the pre and post clinical evaluations of the participants has been presented in [50].

### Volitional control using brain-machine interface

The volitional control was implemented by monitoring the brain activity related to movement intentions. A BMI was developed to record the brain activations over the motor cortex in order to identify when the patients wanted to move their leg. When these intentions were decoded, they generated a trigger for initiating the movement of the orthosis.

The setup of the BMI consisted of a commercial g.Tec system (g.Tec GmbG, Graz, Austria) with 2 amplifiers and 32 EEG electrodes placed over the scalp, according to the international 10/10 standard. The EEG amplifiers were carried by the subjects in a backpack, and connected to a laptop which processed the neural signals. The processing was done with custom-made C++ software, integrated with Matlab scripts.

The BMI movement intention decoder was calibrated using previously recorded data before starting the closed loop operation of the system. The patients were asked to stand between the parallel bars, wearing the exoskeleton blocked in a resting position, and to rest or to try to move their right leg upon auditory cues. They performed 60 repetitions of rest and movement attempt, and the EEG signals recorded during those periods were used to estimate the brain patterns that controlled the movement onset of the orthosis.

As features to decode these movement attempts, we used the EEG electrodes placed over the sensorimotor cortex (i.e., FC3, FCz, FC4, C3, C1, Cz, C2, C4, CP3, CP1, CPz, CP2, and CP4) and computed the event-related desynchronization (ERD) of the sensorimotor rhythms and the movement-related cortical potentials (MRCP). These are two well-studied correlates of movement that can be used to estimate the onset of a movement with a good temporal precision [51], even in patients with SCI [52]. Therefore, every time that the BMI decoded a movement intention command, it sent a trigger to the finite-state machine (FSM), which used it to move the orthosis when appropriate.

### Adaptive control with Exoskeleton H1

**Fig. 3:**
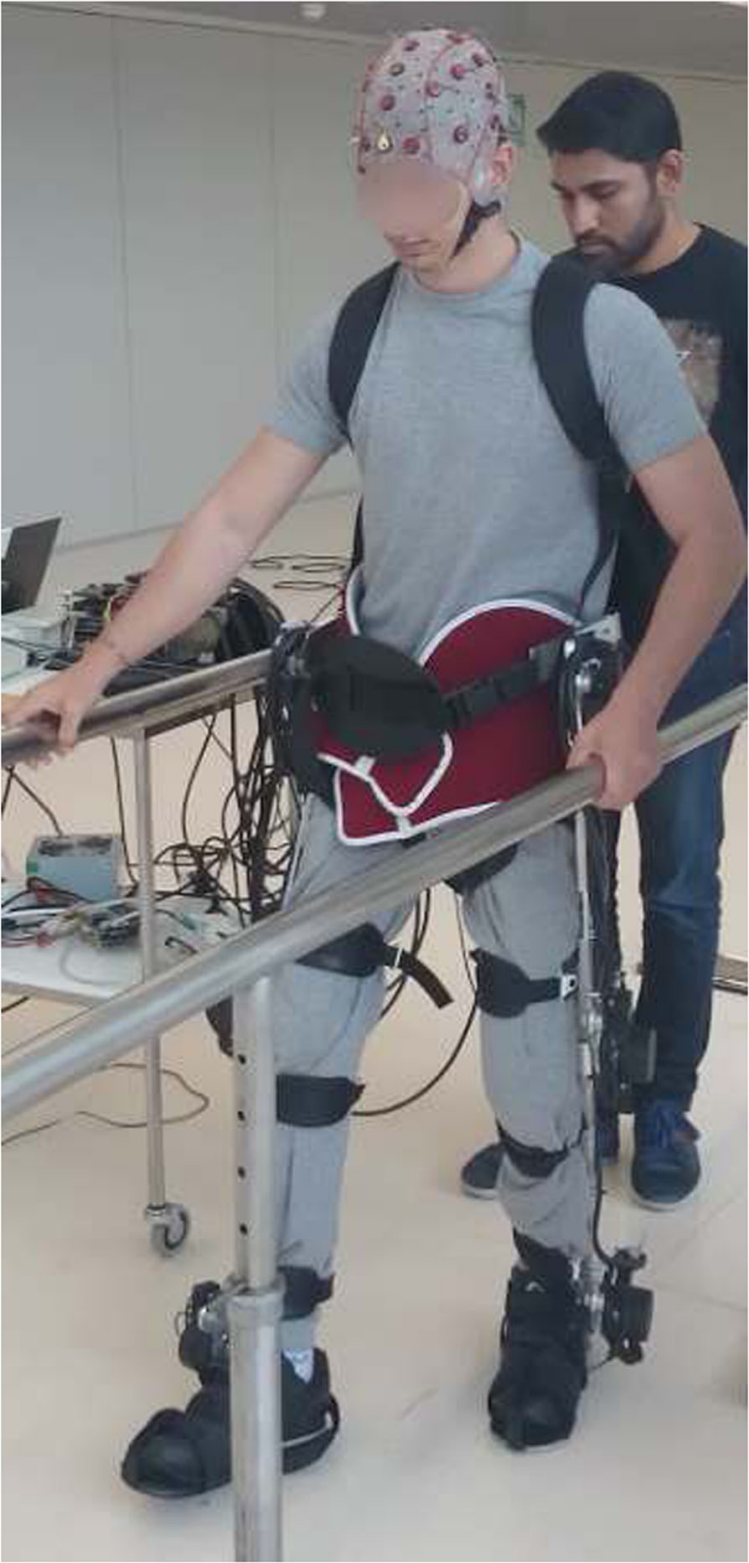
Experimental Setup with an incomplete SCI patient wearing the Exoskeleton, H1, and BMI system

The proposed adaptive control approach for gait assistance is evaluated using H1, a lower limb exoskeleton. H1 is a 6 DoF (degree of freedom) wearable lower limb orthosis with an anthropomorphic configuration to assist individuals with incomplete SCI or Stroke. The exoskeleton, shown in Fig. 3, has been built within the framework of the Hyper* project. H1 has three joints for each leg: hip, knee and ankle, with each joint powered by a DC motor coupled with a harmonic drive gear. The exoskeleton is equipped with potentiometers and strain gauges to measure the joint angles and human-orthosis interaction torques on the links respectively. The exoskeleton permits a stiffness value within the range of 1−100*Nm*/*deg*. A low stiffness value (<10*Nm*/*deg*) will not cause any significant effect on the user’s performance. Similarly, a high stiffness value (>80*Nm*/*deg*) will provide a completely assisted movement, with few or no input from the user [53]. A detailed description about the hardware components and functioning can be studied in [28].

### Finite-state machine

**Fig. 4:**
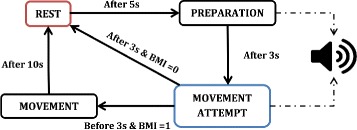
Schematic representation of the FSM-based clinical protocol; the auditory cue is provided in the preparation state and in the beginning of movement attempt state

Event based control approaches are highly appreciated in combinational therapies to make an efficient use of the appropriate tools at different stages of the gait cycle. These event-based gait initiation ensures the real time coordination among the different tools and improves the rehabilitation training methodology [24, 54–56]. Finite state machine (FSM) based BMI correlation with movements are also been used to improve the efficacy of the volitional orders in rehabilitation [40]. In this study, a FSM control model is used to ensure the combination of BMI-exoskeleton and to provide sufficient pause between the different states of the experiment, as shown in Fig. 4. This experimentation is categorized into four states with specific time assigned for each state: Rest, Preparation, Movement attempt and Movement. The time assigned to each state of the clinical protocol was defined based on the previous study with upper limb rehabilitation [57]. Further, the timings of the FSM were decided by our medical team to provide enough time to rest and to perform the task in a safe and effective manner. These timings were specifically designed for the task and population involved in this study.

An auditory cue is provided during the preparation and movement attempt, indicating the patient to concentrate in the movement. The BMI signal is monitored only in the Movement attempt stage, which is further used as the trigger to initiate the movement of the exoskeleton. In any case, if there is no-attempt made by the patient, the machine progresses towards the resting period. Each cycle of the protocol lasts between 20-25 s, including the resting period. The movement time state comprises of the maximum duration (10seconds) needed to complete one gait cycle (2 steps), which further depends on the pace and performance of the patient. Indeed, one gait cycle can be completed within 6seconds. These timings could be modified/ personalized depending on different set-up’s, such as using a robotic exoskeleton with full weight support, or for patients with a higher balance control.

### Evaluation

The gait adaptation strategy presented in “Volitional-adaptive control” section is evaluated by comparing the evolution of the user performance against the application of constant stiffness values.

An adaptive assistance in the joint should assist and motivate the patient to pursue their maximum flexion/extension movement in the joint which also results in a change in behaviour of the interaction torques. Hence, performance in this work is evaluated by monitoring the maximum joint angles and interaction torques. An optimal performance is assumed to produce maximum movement range (flexion/extension) and an increase of the interaction torques in the knee joint. The trajectories of the patients are also recorded to analyse the pattern and freedom of the joint movement.The stiffness variation is also monitored to demonstrate the adaptability of the patient with the orthosis. High stiffness values are expected for joints that need assistance and low values for the rest.

Performance of the presented FSM-based Adaptive control approach is evaluated by monitoring the response time. Response time is given by the onset of BMI trigger and the gait movement initialized. If there is no BMI trigger received in the movement attempt state, it is considered as a trial missed by the participant.

One-way repeated measures ANOVA tests were conducted to evaluate the significance of movement range of motion over the course of three sessions. Statistical significance (*α*) was set at 0.05. A paired t-test procedure with a Bonferroni correction was performed to identify the significant differences between the fixed assistance and adaptive assistance scenario using the exoskeleton. Since two of the participants were not able to complete the total number of 20 trials in session 2, the statistical comparison between the significant differences was performed only with recorded information from session1 and session 3.

## Results

The proposed adaptive control strategy has been tested and evaluated with four incomplete SCI individuals. As a pilot study, this control approach was first evaluated with healthy subjects, without a BMI system [38, 49].

**Fig. 5:**
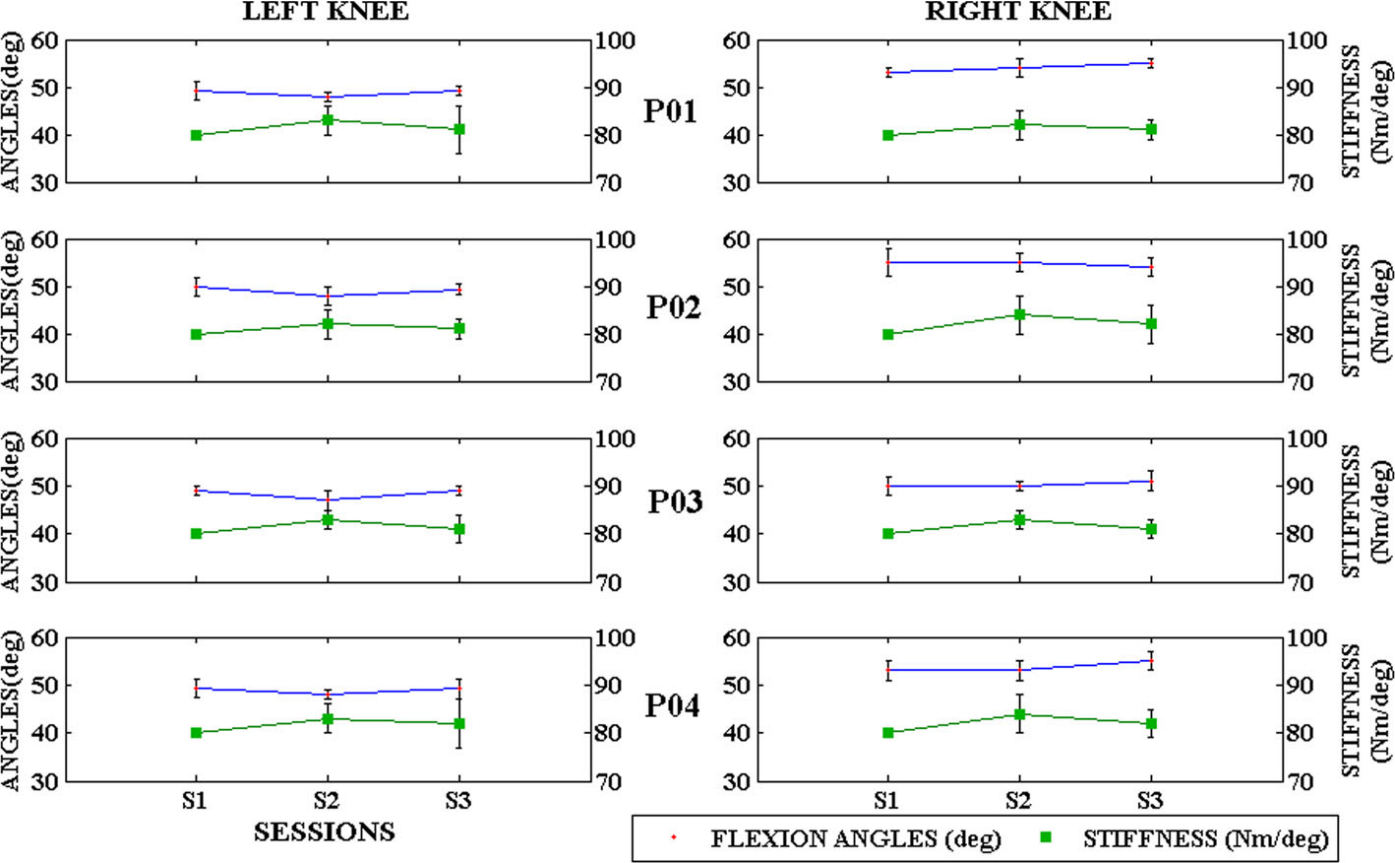
Mean and Standard deviation of the knee flexion angles and Stiffness adaptation observed in both legs of the 4 patients in each session (S1, S2 and S3); Patient P04 had a high sensibility in both the legs which resulted in a similar range of assistance after both sessions. Note that in session 1(S1), a fixed stiffness of 80Nm/deg was used for all the patients

**Fig. 6:**
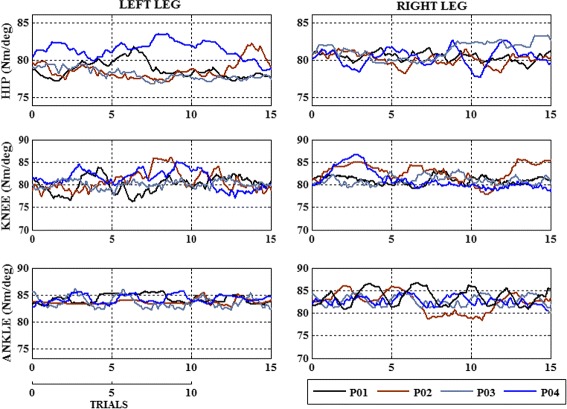
Stiffness adaptation performed for each patient, for 15 gait trials, in session 3. The hip joints showed minimum variations in comparison with the knee and ankle joints, because of the lateral compensation

The efficiency of the adaptive assistance provided by the control model is evaluated for every patient in comparison with their individual gait pattern, obtained by applying the fixed stiffness. The maximum and minimum knee flexion angles obtained at the end of each session is taken and the mean and standard deviation value within n gait cycles of each session is considered for the analysis of patient evolution, as illustrated in Fig. 5. This maximum and minimum flexion angles evaluates the adaptation performed by considering the patients movement in their optimal conditions and in presence of fatigue. The stiffness adaptation performed for each user, for knee joint, is observed within a range of 80±5*Nm*/*deg*, as shown in Fig. 5. The stiffness relaxation is observed more in the session 3, indicating the exoskeleton’s adaptation to the users’ movement. Similarly for the hip joint 80±2*Nm*/*deg* and the ankle joint 82±4*Nm*/*deg*, Fig. 6. The hip joints showed minimum variations in comparison with the knee and ankle joints, because of the lateral compensation. High stiffness variation is observed in the knee joint, as a result of the flexion/extension movement performed, but also stabilized within a range at the end of 10 to 15 trials. The hip and ankle joint showed a minimum stiffness variation in comparison with knee joint, because of its biomechanical properties.

Table 2 illustrates the mean and standard deviation of the stiffness variations and knee flexion angles observed over the course of three sessions. The response time and number of gait trials performed is also presented to evaluate the significance of the FSM based control model in gait training. The number of misses and gait trials performed are evaluated based on the gait triggers received within the movement attempt state. Response time evaluates the efficacy of the FSM based control approach in a volitional based adaptive control model. Lower time stamps indicate that the user has initiated the movement within the movement attempt state.

**Table 2:** Detailed analysis of the results obtained with the FSM-based control model

Patient ID	Session	Gait trials	Number of	Stiffness (*Nm*/*deg*)		Flexion angles (deg)		Response time ^*b*^
		performed	trials missed ^*a*^	LK	RK	LK	RK	(s)
	1	20	0	80	80	49±2	53±1	-
P01	2	24	10	83±3	82±3	48±1	54±2	0.82±0.66
	3	24	12	81±5	81±2	49±1	54±1	0.85±0.63
	1	40	0	80	80	50±2	55±3	-
P02	2	2 ^*c*^	0	83±3	84±4	48±2	55±2	0.92±0.24
	3	25	4	82±5	82±4	50±1	54±2	0.66±0.41
	1	27	0	80	80	49±1	50±2	-
P03	2	7 ^*c*^	0	83±2	83±2	47±2	50±1	0.89±0.14
	3	23	8	81±3	81±2	49±1	51±2	0.93±0.76
	1	32	0	80	80	49±2	53±2	-
P04	2	9	13	83±3	84±4	48±1	53±2	1.18±0.25
	3	19	10	82±5	82±3	49±2	55±2	0.81±0.56

LK- Left Knee; RK - Right Knee

Mean = averaged across the session, SD = Standard deviation

Session 1 was performed with the Fixed stiffness and Manual trigger; Session 2 & 3 with variable stiffness and BMI trigger

^*a*^Number of trials missed = Total trials-gait trials performed

^*b*^Response time = Onset of BMI trigger- Movement Attempt state

^*c*^Trials were interrupted due to temporal restrictions of the patient

**Fig. 7:**
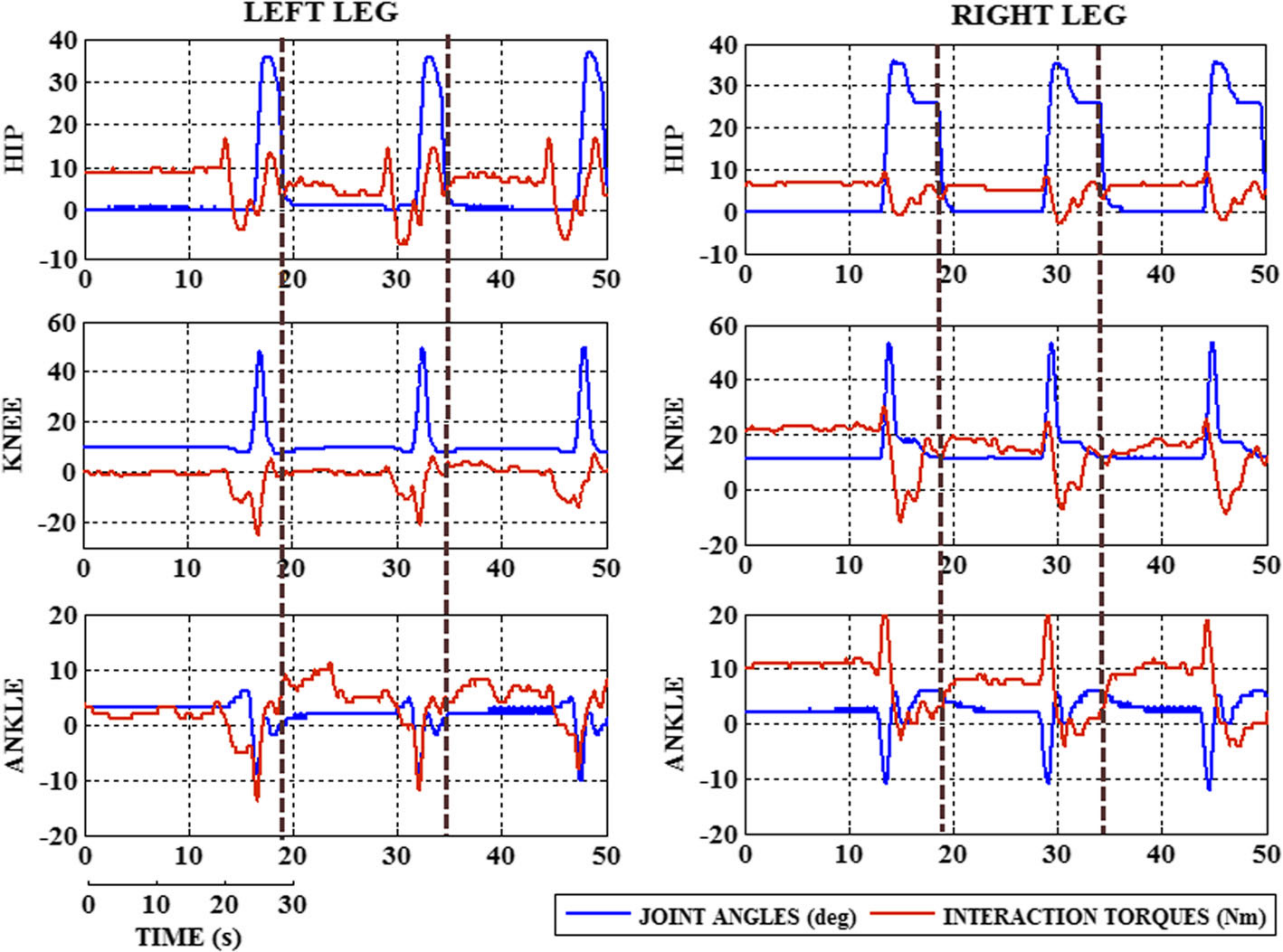
Joint trajectories and interaction torques of the patient P01, with fixed stiffness (80Nm/deg); Note: Joint stiffness is increased to 90 Nm/deg at the end of each gait cycle, indicated by brown line

The results of the Patient P01 are used to explain the volitional-adaptive control and its effect in the patient performance. The joint trajectories and interaction torques of the patient in the fixed stiffness session is presented in Fig. 7. The interaction torques of the left leg is found lower and in negative direction, due to the complete assistance provided by the exoskeleton, for instance at time *t*=32*s* its −20 *Nm* and −12 *Nm* in the knee and ankle joints respectively. The right leg showed more positive interaction torques, +20 *Nm* in knee and +20 *Nm* in ankle at *t*= 30 *s*. At the end of each gait cycle, the stiffness of the joints is increased to 90 *Nm*/*deg* which helps the patient to keep their knee joint rigid, to maintain the equilibrium position.

**Fig. 8:**
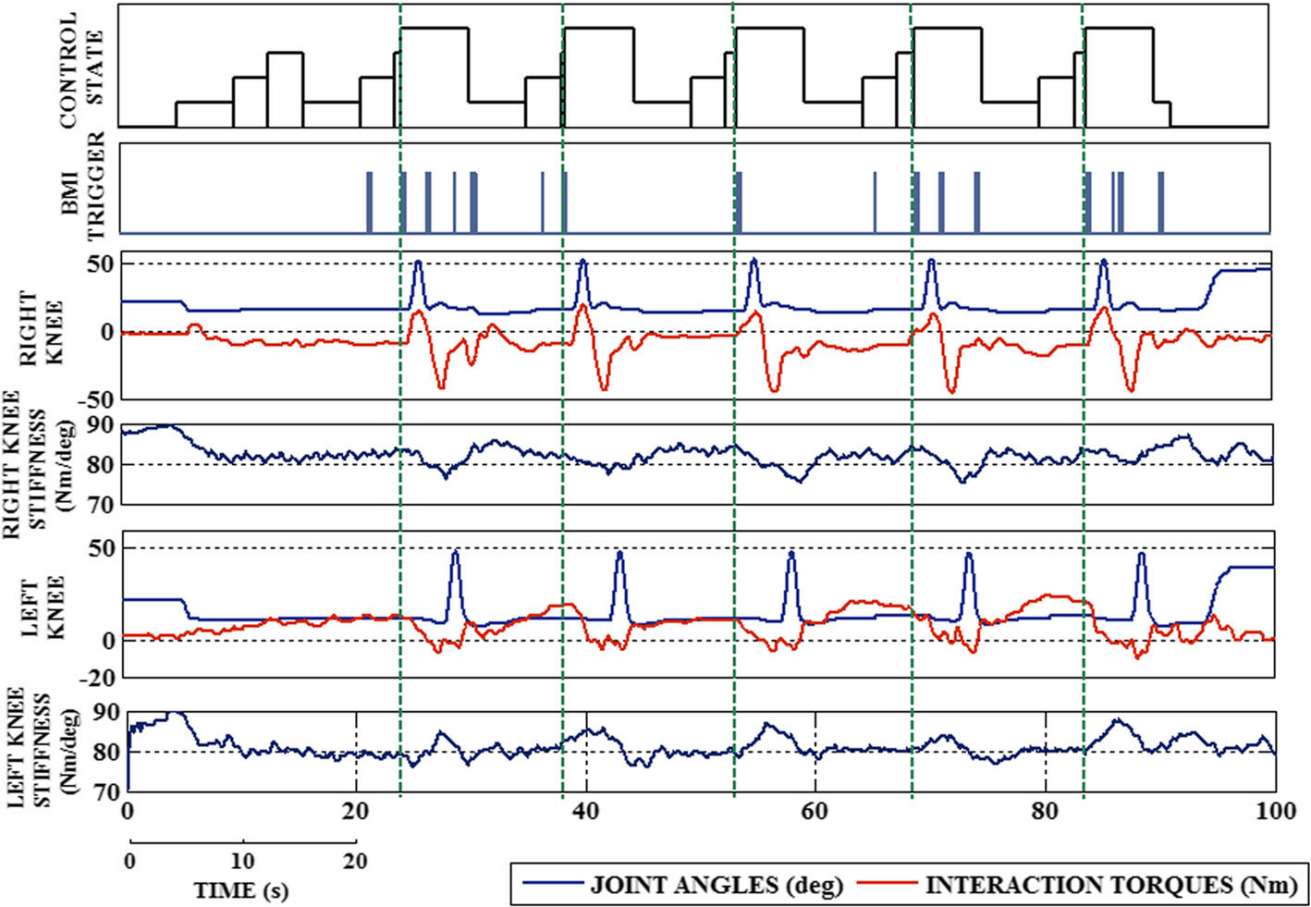
Joint trajectories and interaction torques of the patient P01 with adaptive stiffness; Gait initiation occurs with the onset of BMI trigger (green line) at the beginning of each gait cycle. BMI triggers received at the movement attempt stage are only considered for the gait initiation. The control states indicate the transition between different states indicated in the FSM and the BMI trigger received at the movement attempt stage

In case of the variable stiffness, the interaction torque is initially observed low and the angles are also within a similar range, as shown in Fig. 8. After a few series of trials, the interaction torques in the left leg gradually increased due to the influence of the adaptive stiffness, motivating the patient to pursue the movement (Fig. 8), which subsequently resulted in gradual increase in the knee flexion movement. For instance at time *t*=30 *s*, a maximum torque of 12 *Nm* is observed which gradually increases to 24 *Nm* at *t*=80 *s*, even within after series of 5 gait trials. A similar change in the interaction torques is observed in the hip and ankle joints. The predefined gait pattern is used to guide the patient movement and not impose/force to follow the desired trajectory. Different maximum knee flexion movement in both legs shows the independent stiffness adaptation performed. The participants showed an effective improvement in their performance, which can be identified by the different maximum knee flexion in both legs.

**Fig. 9:**
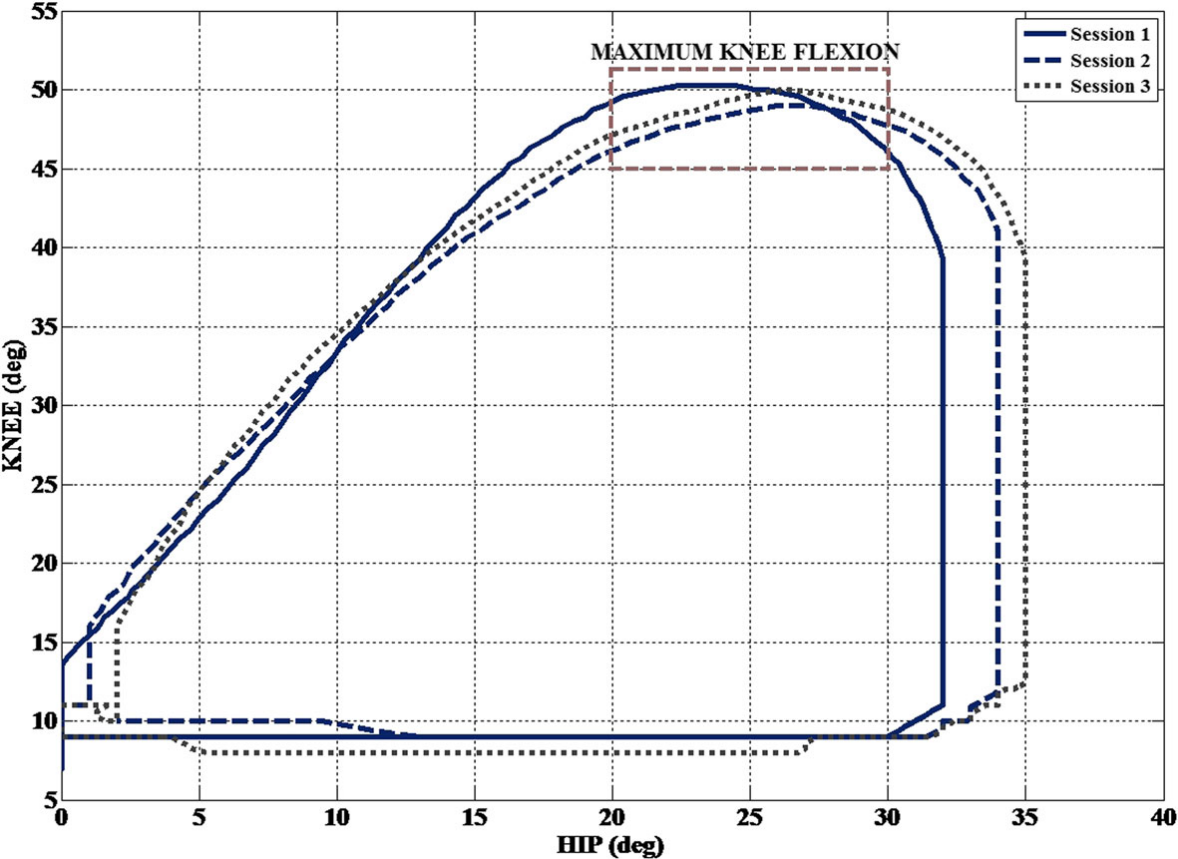
Maximum flexion of Left knee of the patient P01, at the end of each session; Note: Session 1 was performed with fixed stiffness and Sessions 2 and 3 represents the adaptive assistance provided

The stiffness value of the knee joint is different in right and left leg because of the performance and interaction of the user. The minimum variation and higher stiffness value in the ankle joint stiffness is a result of the biomechanical property of the ankle joint, withstanding joint position to maintain equilibrium. The resulting maximum flexion from the fixed and adaptive stiffness is compared to demonstrate the adaptive behaviour and assistance of the exoskeleton, Fig. 9. The maximum flexion of the knee joint coincides with the change in the hip trajectory which explains the compensatory movement performed by the patient.

A repeated measures ANOVA with a Greenhouse-Geisser correction determined that there is significant difference in the movement range of motion among the three sessions *F*(1,19)=22.814,*p*<.0005. Paired t-test with Bonferroni correction determined that there is statistically significant difference in the movement range of motion within the three sessions *t*(19)=−4.776,*p*=.0009. Further, individual subject performances were also analysed and a significant difference (*p*<0.05) was observed for all the subjects except for patient P04 (*t*(25)=3.419,*p*=.076). Therefore, we can conclude that an adaptive control model can improve the gait training in comparison with a fixed assistance approach.

## Discussion

A volitional-based adaptive control model for performing gait training with a wearable exoskeleton has been tested and evaluated with four incomplete SCI individuals. The paper elaborates the first evaluation of the presented control model, using the interaction torques, with patients with motor dysfunction. The adaptive control model introduced in this article provides sufficient assistance to the patient to perform the movement along with ensured active participation. The active participation of the patient is needed throughout the therapy to make an effective contribution out of therapy [58–60]. In this work, active participation is ensured by the inclusion of the human-orthosis interaction torques in the adaptive control model, along with the trajectory deviation. Further, the motor related neural activity of the patient, identified by using the BMI system, is used as a trigger to initiate the gait movement. Both the human-orthosis interaction and neural activity can be deemed as the volitional orders from the user, which in turn helps to avoid slacking. This combination of therapeutic tools, to monitor the neural activity and joint activity, motivates the patient to pursue the therapy with high diligence and helps in improving their movement [61].

The use of a FSM model ensured that the output from the BMI system is accessed only in the gait initiation stages and to avoid the interruptions of the neural signals throughout the gait movement. The FSM model disregards the BMI triggers which are received outside the movement attempt state, thus ensuring the safe and effective use of the combined tools. The onset of the BMI trigger initiates the gait movement followed by the stiffness adaptation (Fig. 9). The time lapse between the onset of BMI trigger and the gait initiation is observed to be <2*s*, which evaluates the real time response of the system. The efficient BMI tracking followed by the gait initiation response was helpful in motivating the patient to pursue the movement and further helping them to avoid slacking.

Adaptive control approaches help in providing the suitable support to the user in real time and to ensure stability by maintaining high endurance in specific joints. The design of such adaptive control models helps in choosing the specific action to be performed in each joint whilst maintaining synchronization among the other joints. The synchronization among the joints is another way to ensure the stability but also involves imposing a trajectory to be followed in each joint [62]. Several existing models of adaptive controllers are capable of providing the necessary arrangements in the rehabilitation scenario [7, 63, 64]. Stiffness based adaptation are capable to provide efficient solutions with multiple combination of devices. The use of adaptive stiffness can be deemed as a bio-inspired strategy [54], due to the existence of a similar mechanism in healthy walking individuals. The use of stiffness based approach helps in maintaining the equilibrium, keeping the knee joint rigid, whilst providing sufficient assistance to the movement.

A control model based on fixed stiffness value cannot provide sufficient assistance or resistance, with respect to the movement. In case of a fixed high stiffness, the function of the exoskeleton is almost similar to that of position control; thus the error with respect to the reference trajectory will be the minimum. Similarly, with high fixed stiffness, the patients demonstrated high interaction torques (Fig 8), which can be a result of the opposition force applied by the patient or due to some uncomfortable movement performed by the patient, out of their capabilities. For instance, the left leg of the patient P01 was comparatively weak and was completely assisted by the exoskeleton’s movement. This assistance helped the patient in achieving the necessary flexion angles but with the negative interaction torques. Fixed stiffness assists or forces the patient to follow the reference trajectory, but the behaviour of the interaction torques explains the kind of movement pursued by the patient especially in the weaker leg. The maximum knee flexion angle, obtained by applying fixed stiffness, is used to evaluate the performance of the adaptive stiffness approach. Simultaneously, the interaction torque is monitored to analyse the efficiency of the real time stiffness adaptation provided by the proposed control strategy.

In the adaptive stiffness case, there is a change in the behavior of the interaction torques (positive direction) as the user was not being forced to perform a trajectory, similarly to other works [24]. The walking trajectory of the patient was not adapted to the unaffected leg, which helps in providing assistance independently, as showed in the case of stroke patients in [25, 30]. In some cases, the patient applied a similar force but could not reach a flexion angle similar to the fixed stiffness mode. The patients applied a compensatory walking by modifying the hip movement with respect to the knee flexion, which challenged the stiffness adaptation to be performed in real time. An increase in the maximum knee flexion movement is observed in both legs, which invariably signify the adaptive assistance provided. Further, the evolution of the stiffness value does not follow a similar pattern for all the joints and converges to a narrow range after a series of trials. The stiffness variation was in coordination with the flexion and extension movements, especially with the knee joint. The increment in the interaction torques (towards positive direction) demonstrated the efficiency of the adaptive stiffness approach, which helps the patient to perform a movement in the weaker leg. This joint stiffness variation results in exerting an assistive or resistive behaviour, depending on the direction of the movement, similar to the flexion-extension movement of the joint. All the patients, except for P04, demonstrated an increase in the knee flexion movement, at the end of two adaptive stiffness sessions. The patient P04 was reported with a high sensibility in both the legs, which might be a reason for the no-significant change in behaviour of the joint flexion limits.

The stiffness adaptation provided to the patients was based on the assigned individual confidence factor. The confidence factor helps in moderating the assistive behaviour and to ensure that sufficient assistance was provided, subjective to the patients’ individual performance. Based on the preliminary studies conducted with healthy subjects [38, 49], we can emulate the influence of the lower to higher confidence factors in the stiffness adaptation provided. In this work, a constant confidence factor (0.6) was considered for all the patients because of the similar level of impairment and performance. An interesting property of this control approach is that the confidence factor can also be modified during a therapy as a parameter that evolves with the progress in recovery of the patients. In case of healthy subjects the confidence factor was assumed to be 0.9 and the stiffness adaptation was observed in a minimum level such as to provide less assistance [49].

In this work, the hip joint of all the patients, showed a little variation in the joint stiffness and more adaptable behaviour in real time without imposing a trajectory. In most cases the subjects tried to compensate the movement by modifying the trajectory of the hip joint which helped in realizing increased knee flexion movement. This compensatory movement is observed even in normal walking as a consequence of muscle weakness, as presented in [65]. The stiffness variation for the hip joint was observed lesser than the variation in the knee and ankle joint. This might be due to the lateral movement of the user’s hip joint which compensates the joint trajectory, as other works have shown in [66, 67]. The lateral hip movement helps in maintaining the equilibrium and stability. Since the exoskeleton H1 is a planar robot, the lateral hip movement cannot be monitored; however this orthosis limitation does not affect the proposed control strategy. The interaction torques, in case of the adaptive stiffness, showed a major variance in comparison with the fixed stiffness output. This was obviously due to the sufficient assistance provided to the weaker legs, compared to the leg with better mobility. The leg trajectory showed a progressive change as a result of the increase in the interaction torques. The flexion and extension movement of the knee joint is essential in walking for maintaining the transition between gait phases. This repetitive movement in the knee joint also reflected in the stiffness adaptation performed, which is observed to stabilize after a few gait trials.

The use of BMI in rehabilitation therapies is a step forward towards combining individual tools to ensure an advancement in therapy, which is deemed as a top-down approach [32]. These BMI based systems have proved to improve the motor cortex ability of physically impaired patients while being trained for a long-term [68–70]. In the present study, the BMI system is used as a volitional order to trigger the gait movement, ensuring the user involvement in therapy and to train the motor cortex [71]. It allowed processing the brain activity of the subjects in order to detect when they wanted to move, and start accordingly the movement of the exoskeleton. On average, the BMI detected correctly the movement intention of the SCI subjects in 77.61±14.72*%* of the trials they performed [51]. These performance values are in line with the current state of the art in BMI-based movement intention decoding with SCI patients, which are generally lower to the values achieved by healthy subjects due to the higher difficulty of performing the task for patients with poor balance, and to the differences in the cortical activity that difficult the detection of their motor commands with BMI’s [72]. These results are relevant as they were obtained in a realistic environment, with special emphasis in the safety and usability, and involving a population of patients what could potentially benefit from this type of rehabilitative intervention [51].

Although, the current volitional-based adaptive control model has been evaluated with four SCI individuals, the results obtained are convincing in terms of improved free range of motion in the knee joint and interaction torques in all the joints. This feasibility study helped in convincing that the control approach is favourable in terms of appreciating the residual motor development in SCI individuals. The current state of the art in non-invasive BMI’s does not allow a fine control in noisy environments, or in different tasks such as turning to different directions or stopping, mainly due to the contaminations that affect the EEG signals due to the motion of the subjects (i.e., artifacts). On the other hand, the adaptive control studied and evaluated in the present study is independent of those aspects, and it modifies the impedances to adapt the joints’ trajectories in real-time, regardless of the phase of the gait or other external factors. A detailed study with a longer period of training and continuous practice with the wearable exoskeleton will help in describing the control approaches efficiency and also make it viable for a wider group of participants.

## Conclusion

A volition-based adaptive walking strategy has been evaluated in function of the position and human-orthosis interaction torque, thus ensuring a beneficial and safe therapy. The strategy has been evaluated and tested with four incomplete spinal cord injury individuals. The volitional commands from the BMI system, by monitoring the motor activity of the patient, are determined at the beginning of each gait cycle. The experimental results showed that the user’s gait intention was recognized effectively and followed by the leg movement. Similarly, the stiffness value of each joint adapts dynamically to the user needs and keeps the joint positions bounded within the limits of the reference gait, in real time. The interaction torques of the weaker leg gradually increased over the course of the trials also maintaining the same joint positions limits, evidence of the user motivation. The wearable robot was tested with no body weight compensation, which shows the reliability of the control strategy for ensuring the minimum dynamic stability needed for the experiments using handrails, in presence of ground reaction forces. The performance of the proposed control method was evaluated by comparing the results of the adaptive assistance against fixed stiffness trajectory.

## Abbreviations

ASIA: American spinal injury association
ANOVA: Analysis of variance
BMI: Brain-machine interface
EEG: Electroencephalography
ERD: Event-related desynchronization
FSM: Finite-state machine
MRCP: Movement-related cortical potentials
SCI: Spinal cord injury
